# Cosmogenic ^22^Na, ^7^Be and terrestrial ^137^Cs, ^40^K radionuclides in ground level air samples collected weekly in Kraków (Poland) over years 2003–2006

**DOI:** 10.1007/s10967-014-3049-6

**Published:** 2014-03-09

**Authors:** Sylwia Błażej, Jerzy W. Mietelski

**Affiliations:** The Henryk Niewodniczański Institute of Nuclear Physics PAN, Radzikowskiego 152, 31-342 Kraków, Poland

**Keywords:** Cosmogenic radionuclides, Radioactivity of air, ^22^Na, ^7^Be, ^137^Cs, ^40^K, Aerosol retention time, Low background gamma spectrometry

## Abstract

A low background gamma spectrometer with an Etruscan, 2500 years old lead shield and a muon veto detector were applied to study ^22^Na and ^7^Be activity concentration in ground level air aerosol samples collected weekly over the years 2003–2006 in Kraków. Each sample was formed with ca 100 000 m^3^ of passed air, collected with two parallel ASS-500 high volume air samplers. The results for ^40^K and ^137^Cs are also presented for reference and comparison. Presented frequency distributions for activity concentration and correlation between the obtained results are discussed. The activity concentration results confirmed seasonal variation of activity to be different for all the investigated radionuclides. Moreover, the seasonal variation in nucleus activity ratio was also noticed for ^22^Na and ^7^Be. Cosmogenic radionuclides being mainly of stratospheric origin, are subsequently attached to fine aerosols, via which they are transported to the ground level air. The mean aerosol transport time within the troposphere was estimated as equal to 7.5 days on average, reaching even 50 days in warm seasons. Limitations of the applied model were identified.

## Introduction

The cosmogenic radionuclides present in the Earth’s atmosphere are produced mainly by secondary cosmic rays in the stratosphere or troposphere, with the relative volume of about 2/3 and 1/3, respectively [1]. The maximum production rate is noticeable at the altitude of approximately 20 km [2, 3]. Spallation reactions caused by high-energy particles on oxygen, argon or nitrogen atoms are one of the important processes [4]. For example, ^7^Be, one of the most active cosmogenic radionuclides, can origin from several nuclear processes such as: ^14^N(p,2α)^7^Be, ^16^O(p,^10^B)^7^Be, ^14^N(n,^8^Li)^7^Be or ^16^O(n,^10^Be)^7^Be [1, 5]. The second cosmogenic nuclide studied here, ^22^Na, is formed mainly in spallation of argon nuclei by a secondary cosmic ray neutron [4]. ^7^Be decays (T_1/2_ = 53.3 days) by electron capture to stable ^7^Li emitting gamma radiation of 477.6 keV and a monoenergetic neutrino. ^22^Na decays (T_1/2_ = 2.602 years) by a beta plus process to ^22^Ne emitting a photon of 1,274.53 keV, a positron and a neutrino. Both nuclides were also formed due to thermonuclear explosions in the atmosphere [6] conducted in the cold war era. Relatively short half-life times of the nuclides made their current activity levels no longer affected by nuclear tests. The production rate is mostly affected by the solar activity with a reverse proportional relation, i.e. the higher solar activity, the lower production rate. Numerous studies [7–14] have reported the relation between ^7^Be activity and the solar cycle. However, the most detailed study was recently published as two papers [5, 15]. Such a reverse proportionality to the solar activity results from additional screening of the Earth’s atmosphere with the magnetic field generated by charged particles from the solar wind trapped in the magnetosphere (so called Forbush effect). The Sun’s activity is basically multi-cyclic, with the main cycle of 11 years. Many parameters characterize the Sun activity; one of such is called the Wolf number and it is a normalized number of black spots on the Sun. Due to the relationship between cosmogenic radionuclides production and the Earth’s magnetosphere, a dependence of production rate of such radionuclides on the geomagnetic latitude is observed. A high production rate at high latitudes drops low close to the magnetic equator. Generated nuclides are attached to fine aerosols present at high altitudes and transported along to the ground level air [16]. The effectiveness of such a transport process depends on many factors; in the temperate climate zone one of the most important ones is the temperature gradient in the troposphere, which governs the air convection. This vertical movement of air masses is much more intense in warm seasons than in the cold ones. The resulting seasonal variation of ^7^Be is a well known phenomenon [17–19]. The typical ground air activity concentration for ^7^Be corresponds to few mBq/m^3^, whereas the activity concentration for ^22^Na falls below 1 μBq/m^3^. This explains why ^7^Be activity concentration in air happens to be the subject to numerous reports, which it is not the case for ^22^Na [20]. Contemporary detection of ^22^Na faces difficulties. Still, both ^7^Be and ^22^Na used to be applied in modelling the transport of aerosols from the stratosphere to the ground level air [4, 21, 22] as an alternative to more typical usage of ^7^Be and ^210^Pb [23, 24]. Recently, the progress on low-background gamma spectrometry achieved mostly due to application of anticoincidence active shields or digital coincidence systems, resulted in the “renaissance” of new studies on ^22^Na [15, 25, 26].

Many atmospheric processes affect radionuclides concentration in air. A number of studies reported the relationship between the precipitation yield and the level of the observed activity of cosmogenic radionuclides. Rain effectively cleans the air from aerosols, hence from cosmogenic radionuclides. In general, the observed level of cosmogenic radionuclides, based mainly on ^7^Be studies, depends also on the exchange of air between the stratosphere and troposphere, particularly including springtime injections of the stratospheric air into the troposphere [1, 14, 27–31]. Apart from this depends on the vertical thermal movements velocity in the troposphere governed by temperature distribution [32], the precipitation rates [7, 9, 15, 33–37], horizontal atmospheric movements of air masses from lower to higher latitudes [1, 22, 32] and the atmospheric diffusion [38]. All the processes contribute to seasonal changes in ^7^Be concentrations in ground level air [9, 39], which sometimes “mask” the solar cycle dependence [12, 40]. In Europe springtime increase of ^7^Be concentration in air is typical. In Japan, however, it seems to be accompanied by a similar increase in autumn [41, 42] or even winter [43], that have never been observed in Europe. The paper [46] provided an explanation to the peculiarity of Japanese ^7^Be observation [41–45] as resulting from the influence of very cold air blown from Siberia in winters, which clashes with much warmer air masses coming from the Pacific Ocean.

The model proposed in [4] used both ^7^Be and ^22^Na to determine the mean time for the retention of aerosols in the troposphere. One of the goals of our study was to adopt this model to the European data to check its applicability to other areas. We aimed at checking weekly the regularity in ^7^Be and ^22^Na behaviour reported in our previous study for half-a-year samples [19], and we also wished to demonstrate our capability to determine ^22^Na activities on the levels falling below 1 μBq/m^3^, as we apply the spectrometer with a cosmic muons veto detector and 2,500 years old lead in shield. Apart from cosmogenic radionuclides, the activity concentration of two terrestrial nuclides, namely ^137^Cs (anthropogenic) and ^40^K were measured to find similarities and differences with the cosmogenic nuclides. The study was conducted as a PhD project whose selected main findings are presented here.

## Method

### Samples

Samples of ground level air aerosols were collected over the years 2003–2006 at the premises of our Institute in Kraków (Southern Poland, 50.08ºN, 19.92ºE, 240 m a.s.l.) with two high volume aerosol sampling stations produced by CLOR (Polish Central Laboratory for Radiation Protection), Warsaw, type ASS-500. The nominal airflow rate for both ASS-500 is 500 m^3^/h. [47]. They use Petryanov FPP-1.5–1.5 filters. The ASS-500 is a typical aerosol sampler used presently at 12 locations in Poland for routine air radioactivity monitoring. The monitoring network works on weekly basis, collecting aerosols from 50 000 to 80 000 m^3^ of air on a single ASS-500 filter. Our two air samplers are not identical. The filter dimensions of the older model are 0.57 m × 0.45 m, while the other square filter is 0.45 m by 0.45 m [19]. The exposed filters (with the supporting cloth removed) are pressed to 4–5 mm high pellets with a 51 mm diameter. In routine monitoring such filter pellets undergo a whole day measurement with a typical low background germanium gamma spectrometer equipped in a 10 cm lead with copper and cadmium linings, providing a minimum detectable activity for ^22^Na of about 0.7 μBq/m^3^. This enables us to detect ^22^Na in some single filters only during warm seasons, i.e. spring or summer. Here we applied a double filter pellet 51 mm in diameter but about 10 mm high. Each examined sample contained aerosols from about 100,000–150,000 m^3^ of air.

### Measurement system

**Fig. 1:**
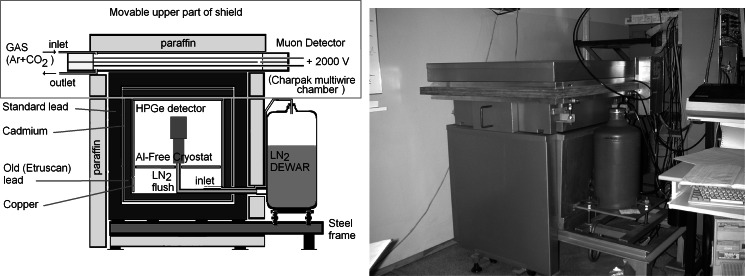
The scheme of the shield (*left*) and the view of ultra low background gamma spectrometer (*right*) applied in our measurements [49]

An ultra low-background homemade gamma spectrometric system (Fig. 1) was arranged for the measurements [48, 49]. It comprised about a 25 % efficiency HPGe detector situated in a stainless steel and a copper U-type aluminium free cryostat, with a carbon composite endcup top window and a shield made of (from inside to outside): 1 cm electrolytic copper, 5 cm Etruscan, 2,500 years old lead, 2 mm cadmium, a standard 10 cm lead and the 10 cm outer steel containers filled with paraffin. An active shield made of a multiwire proportional chamber, served as muon veto detector. 0.44 pulses/second are recorded in the spectrum range from 40 keV to 3 MeV [49]. It was calibrated with a widely available mix multi-gamma source produced by Polatom, Świerk (SZN-3) in a form of a single pressed filter 51 mm in diameter, and about 5 mm high. A trick was performed to circumvent production of a dedicated calibration source. The calibration curve was calculated from the data obtained by adding two calibration source spectra, corresponding to two measurements—one in a standard geometry, and another one performed within the same time but with a plain pressed filter used as a separator. Obviously, while calibrating the spectra were added, time retained, and activity was doubled.

**Fig. 2:**
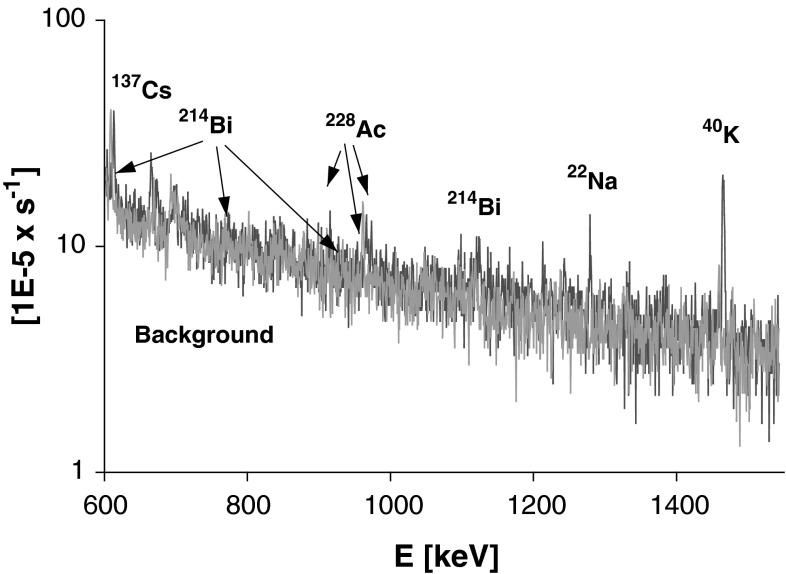
A part of gamma ray spectrum for air filter sample No 38, 2004 (*red*) compared with the spectrometer background (*blue*). (Color figure online)

Each sample, i.e. a double filter exposed for a week, was measured for 7 days. The measurement was performed at least 1 month after collecting the aerosol in order to reduce unsupported activity of short lived radon (^222^Rn, ^220^Rn) progenies, leaving only in both U and Th series supported component for short lived isotopes. A sample spectrum compared to background is presented in Fig. 2. Please note that ^40^K peak is not visible in the presented background, hence ^22^Na peak is practically undisturbed by Compton edge from ^40^K peak.

The minimum detectable activity (using the standard Currie formula, [51] ) for ^22^Na was reduced from about 0.7 μBq/m^3^ in a routine monitoring regime (i.e. a single filter, 1 day measurement, a standard low background spectrometer) by almost an order of magnitude, and fell to about 0.1 μBq/m^3^ for our set-up (a double filter, an ultra low level spectrometer, 7 days measurements).

### Quality assurance

The presented measurements were collected over a period of 4 years. A standard source, i.e. ^137^Cs and ^60^Co, was measured to check stability of the detector efficiency calibration over the time, randomly at first, later regularly once a week, between each of the two measurements. The calibration was also positively double-checked by comparing ^40^K, ^7^Be and ^137^Cs results from the double filter set with the results for the same radionuclides from a routine measurement of a single filter. While conducting the measurements reported here, our laboratory participated in intercalibration runs in the field of gamma spectrometry organized by the IAEA, IRMM and Polish National Atomic Agency, PAA; the results were approved. Soon after finishing the measurements, i.e. since 2008, the laboratory was granted Polish accreditation for gamma spectrometric measurements (ISO 17025).

### Applied model for aerosol time retention

The model for mean aerosol retention time proposed in paper [4] is described with the Eq. 1:

1 $${{R_{\text{a}} = \frac{{P_{\text{Be}} (1 - \exp ( - \lambda_{\text{Be}} T_{\text{s}} ))\exp ( - \lambda_{\text{Be}} T_{\text{t}} )}}{{P_{\text{Na}} (1 - \exp ( - \lambda_{\text{Na}} T_{\text{s}} ))\exp ( - \lambda_{\text{Na}} T_{\text{t}} )}}}} $$

where *R*_a_—^7^Be to ^22^Na activity concentration ratio observed in ground air; *P*—production rate in the stratosphere, index denotes the radionuclides, ^7^Be or ^22^Na, assumed *P*_Be_ */P*_Na_ was equal to 1.1 × 10^3^; *λ*-decay constant, indexes as above; *T*_t_—mean time of aerosol retention in the troposphere (unknown—estimated from the model), *T*_s_—mean time of aerosol retention in the stratosphere (20 days assumed following the original paper [4])

The model concept can be explained as follows. In the stratosphere aerosols are marked with two isotopes of different half-life time, which start to act as a clock. Given their mean activity ratio on the ground level (*R*_a_), with an assumed retention time in the stratosphere and the production rate, the retention time in the troposphere can be obtained by fitting. Recently, this model was applied for initial studies by another Polish team [24].

## Results

### Activity concentration

**Fig. 3:**
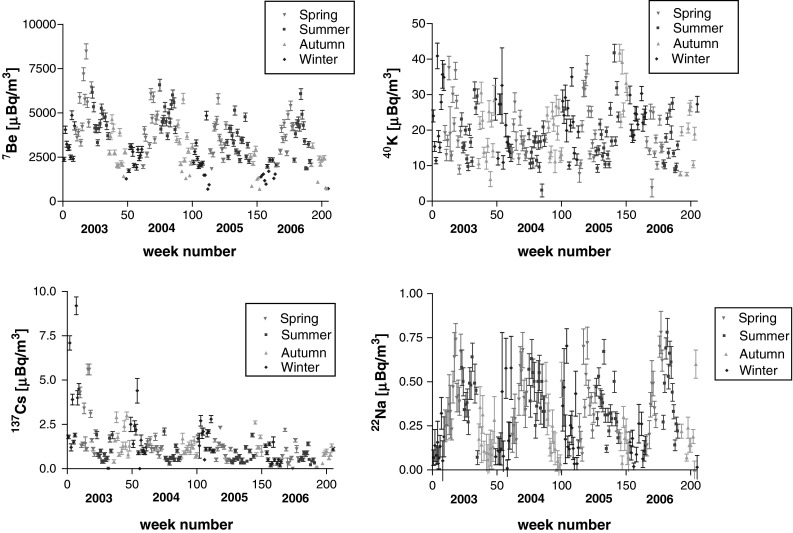
Activity concentration of ^7^Be, ^40^K, ^137^Cs and ^22^Na in ground level air in Kraków in Jan 2013–Dec 2006 for weekly-collected aerosols samples. Conventional four seasons (spring, summer, autumn and winter) are marked with *different symbols*

The results for activity concentration of ^7^Be and ^22^Na, as well as ^137^Cs and ^40^K in the ground level air in Kraków (Southern Poland) obtained weekly over the years 2003–2006 are presented in Fig. 3. All results display seasonal variations. To analyse such a seasonal regularity over the years, the results of each year were divided into “spring” i.e. mid-March, April, May, mid-June (weeks 10–22 of each year), “summer”, i.e. mid-June, July, August, mid-September (weeks 23–35), “autumn” i.e. mid-September, October, November, mid-December (weeks 36–48), and finally “winter” i.e. mid-December, January, February, mid-March (weeks 49–9 of the following year). The mean and median values for activity concentration for all those four seasons of the studied radionuclides are presented in Table 1.

**Table 1:** Mean and median activity concentrations for ^7^Be, ^22^Na, ^137^Cs and ^40^K in ground level air in Kraków, for four seasons over years 2003–2006 [μBq/m^3^]

	Spring	Summer	Autumn	Winter
^7^Be				
Mean	4,058	4,024	2,482	2,280
Median	4,050	4,087	2,379	2,155
^22^Na				
Mean	0.41	0.41	0.16	0.18
Median	0.39	0.40	0.14	0.13
^137^Cs				
Mean	1.44	0.73	1.23	1.80
Median	1.15	0.56	1.08	1.32
^40^K				
Mean	19.5	17.1	22.1	23.6
Median	17.0	16.5	21.8	23.0

For ^7^Be a large periodic spread was observed. Spring 2003 started with the activity concentration of 3,313 μBq/m^3^ and rose up suddenly to 8,470 μBq/m^3^. Over summer the concentration decreased from 6,140 to 3,307 μBq/m^3^. Autumn showed further decrease to the level of 1,420 μBq/m^3^. The lowest concentration of 1,270 μBq/m^3^ was recorded for one winter week of 2003. A similar scheme has been found to repeat for the following years. Surprisingly apart from such a periodic structure, a tendency of slight decrease in time is also observable, which was rather unexpected, since solar activity kept decreasing over these years: the Wolf number (or Annual Sunspot number) decreased from about 60 in 2003 to below 20 in 2006 [5].

The activity concentration of ^40^K in air filters also revealed some seasonal structure. The arithmetic mean was equal to 19.7 μBq/m^3^, with the minimum at 3.0 μBq/m^3^ and maximum at 40.8 μBq/m^3^. The lowest values were observed in summer. The main source of ^40^K is re-suspension of soil particles [50]. This might explain why at summer time, with high vegetation, ^40^K levels are low. High winter levels might come from the local emission—burning wood, biomass and coal for heating [50, 52].

Activity concentration of ^137^Cs in ground level air (Fig. 3) also displays a kind of cyclic structure with minima in late spring-early summer when high vegetation reduced resuspension. Maxima are observed in winters. It suggests that local resuspension cannot be the dominant effect, as Polish winters are characterised with snow covering lands and frozen soil, hence a direct aeolian erosion resulting in high resuspension is very unlikely. However, other local emissions—wood and biomass related burning seems to be responsible for such winter enhancement of ^137^Cs [52] concentration in air. The highest activity concentration of this isotope was recorded in 2003, mean activity for winter was equal to 3.90 μBq/m^3^, ranging from 1.20 to 9.20 μBq/m^3^, which decreased later in spring to a mean of 2.52 μBq/m^3^ with respective ranges from 0.80 to 5.60 μBq/m^3^. Summer mean was equal to 0.98 μBq/m^3^ and it ranged from 0.40 to 1.90 μBq/m^3^, while autumn mean was higher again and ranged between 0.4 and 2.5 μBq/m^3^.

The activity concentrations for ^22^Na have maxima in warm seasons, reaching 0.75 μBq/m^3^ whereas minima close to 0.1 μBq/m^3^ fall within cold seasons. The recorded values correspond with the results reported for a similar period in Switzerland [15] and Finland [26]. The activity concentration determined for other radionuclides were found also comparable to the literature data.

The frequency distributions over all seasons for ^7^Be activity concentration in ground level air in Kraków is bi-modal, with median or mean values close to 4,000 μBq/m^3^ for spring and summer, whereas for autumn and winter to about 2,300 μBq/m^3^. Frequency distributions for ^22^Na in ground level air also show at least bimodal distribution. Despite a bit unclear shape, due to relatively large uncertainties, the means are close to medians and both are more than twice higher for warm seasons (spring, summer) than for the cold ones (autumn, winter). On the contrary to both cosmogenic radionuclides the frequency distribution of activity concentration ^137^Cs is asymmetric in shape, with the mean from 20 to 50 % higher than the median. Another contrasting features—with regards to cosmogenic radionuclides—are summer minima and winter maxima for activity concentration. Frequency distribution for ^40^K activity concentration in ground level air seems rather polymodal.

### Correlations

**Fig. 4:**
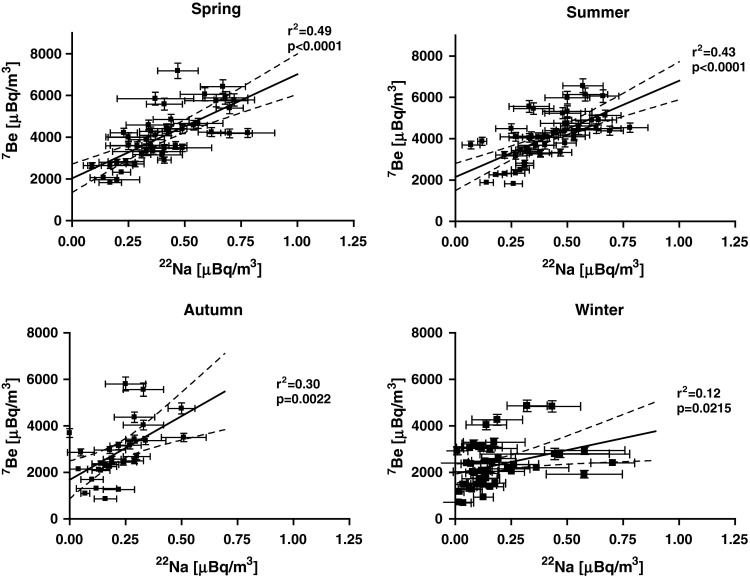
Correlation plots for ^7^Be and ^22^Na activity concentration in ground level air in Kraków in years 2003–2006

High correlation can be expected for activity concentration of radionuclides of similar origin: cosmogenic (^7^Be, ^22^Na) and terrestrial (^137^Cs, ^40^K). Table 2 presents the correlation between activity concentration for all four radionuclides and four seasons. The correlation between ^7^Be and ^22^Na (see also Fig. 4) was the highest for spring (squared Pearson factor *r* ^2^ = 0.49, significance level *p* < 0.0001) and only slightly lower was for summer (*r* ^2^ = 0.43, *p* < 0.0001), whereas it was much lower for autumn (*r* ^2^ = 0.30, *p* = 0.0022), and the lowest value was found for winter (*r* ^2^ = 0.12, *p* = 0.0215). The correlation plot (Fig. 4) for winter might suggest at least two correlation lines to be present: one with a slope of about 6 × 10^3^ (similar to that of warm seasons), and the second one, much stepper with 2 × 10^4^.

**Table 2:** Squared Pearson correlation factors (*r* ^2^—first number in each cell) and significance levels (*p*, second number in cells) for the observed ground level activity concentration in given seasons

	^7^Be	^40^K	^137^Cs	^22^Na
Spring (*r* ^2^; *p*)				
^7^Be	1	0.245; 0.0005	0.246; 0.0005	0.49; <0.0001
^40^K		1	0.192; 0.0004	0.154; 0.007
^137^Cs			1	0.029; 0.246
^22^Na				1
Summer (*r* ^2^; *p*)				
^7^Be	1	0.028; 0.352	0.215; 0.0004	0.43; <0.0001
^40^K		1	0.374; <0.0001	0.003; 0.712
^137^Cs			1	0.072; 0.400
^22^Na				1
Autumn (*r* ^2^; *p*)				
^7^Be	1	0.053; 0.097	0.018; 0.516	0.30; 0.0022
^40^K		1	0.346; 0.0010	0.018; 0.549
^137^Cs			1	0.004; 0.222
^22^Na				1
Winter (*r* ^2^; *p*)				
^7^Be	1	0.115; 0.020	0.117; 0.022	0.12; 0.022
^40^K		1	0.0613; 0.0933	0.066; 0.086
^137^Cs			1	0.033; 0.222
^22^Na				1

For clarity reasons only half of the table is presented

The majority of ^137^Cs observed in ground level air are expected to originate from resuspension and burning of biomass (mostly in winter). However, one cannot exclude that some ^137^Cs can appear in ground level air introduced from the stratosphere. The proportion between them might be governed by such factors as the volume and kind of precipitation, temperature, or the tropopause thickness [53, 54]. Thus one can assume two components for ^137^Cs, one dominant terrestrial, well correlated with ^40^K, and a stratospheric one, correlated with cosmogenic nuclides (^7^Be or ^22^Na). Correlation between ^137^Cs and ^7^Be is rather weak, the highest in spring (*r* ^2^ = 0.246, *p* = 0.0005) and only slightly lower in summer (*r* ^2^ = 0.215, *p* = 0.0004). It is much lower in winter (*r* ^2^ = 0.117, *p* = 0.022), and no correlation at all was found during autumn (*r* ^2^ = 0.018, *p* = 0.516). Similarly, no correlation between ^22^Na and ^137^Cs was observed. It suggests that even some ^137^Cs still comes from the stratosphere, it is much less intense than the one of terrestrial origin. The clearest correlation between ^137^Cs and ^40^K was noticed for summer (*r* ^2^ = 0.374, *p* < 0.0001) and autumn (*r* ^2^ = 0.346, *p* = 0.001). It was much lower for spring (*r* ^2^ = 0.192, *p* = 0.0004), and the lowest for winter (*r* ^2^ = 0.0613, *p* = 0.0933). The winter samples are the most puzzling for ^137^Cs, as they reveal lack of correlation between ^40^K and ^137^Cs, accompanied with the highest values for ^137^Cs. It cannot be the cosmogenic component, as for cosmogenic radionuclides the minimum is observed in cold seasons, so the emission from burned biomass, namely wood, seems to be the most likely explanation [52]. We know from other studies [55] that in Kraków winters display the lowest activity concentration for plutonium in air, with relatively highest ^238^Pu/^239+240^Pu ratio, which might suggest sea-spray from the North Sea (or other spent nuclear fuel) to be an important source of radionuclides in winter. However, the massive sea spray should increase ^40^K, what is not observed. ^40^K is present in the air not only due to soil resuspension and ashed biomass (regular heating for houses), but also due to industrial air pollution rich in minerals. Since this fraction is relatively high and it increases only slightly in cold seasons due to releases from large heating centrums, it stabilises the scale of potassium seasonal variations making it poorly correlated with ^137^Cs. Similarly to ^137^Cs, no correlation between ^40^K and both cosmogenic radionuclides (^22^Na, ^7^Be) was noticed for the ground level air in Kraków in all the examined seasons, except for a weak correlation observed in spring. However, this correlation between ^40^K and cosmogenic radionuclides noticed for spring (for ^22^Na: *p* = 0.0070 and for ^7^Be: *p* = 0.0005) seems to be apparent—the enhanced ^40^K is likely to originate from pollens or resuspension of soil due to agricultural works in the fields, whereas cosmogenic radionuclides are of stratospheric origin.

### ^7^Be/^22^Na activity ratio for weekly samples and the resulting aerosol retention time

**Fig. 5:**
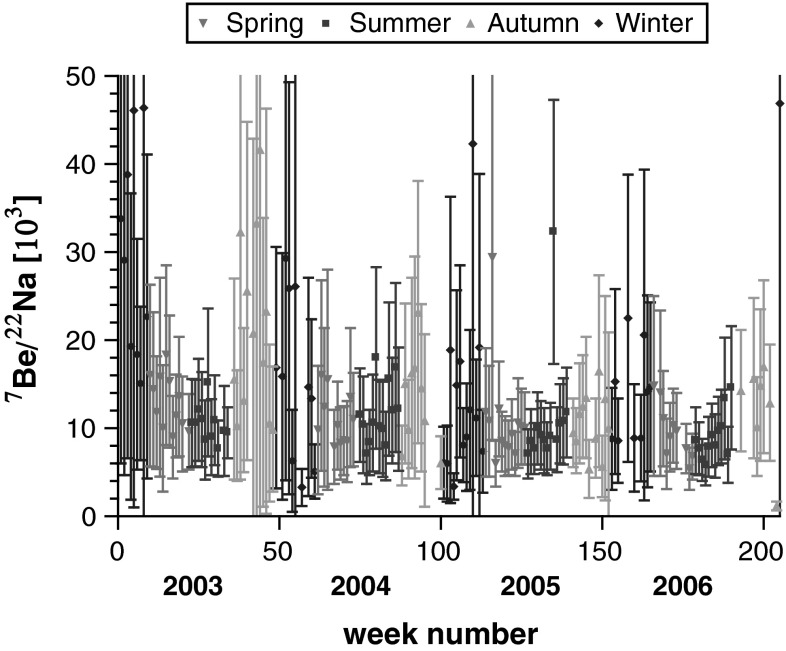
Activity ratio for ^7^Be/^22^Na in ground level air collected weekly in Kraków over 2003–2006. Periodic structure is clearly visible with higher values for cold seasons

The observed activity ratio for ^7^Be/^22^Na in ground level air measured on weekly basis shows a cyclic, seasonal structure with warm periods (spring or summer) maxima and minima in winter or autumn (Fig. 5). It confirms our previous findings [19] where half-year sets of samples were studied and a seasonal variation of ^22^Na to ^7^Be was suggested. Such a variation of cosmogenic isotopes activity ratio was observed and discussed in recent papers from Finland (26) and Switzerland (15). The Finnish paper explains such features by a relationship with global meteorological parameters such as North Atlantic Oscillation, whereas the latter one provides explanation based on the introduced mixing cycle starting with spring injection of cosmogenic nuclides from the stratosphere, summer intense downward mixing within upper troposphere, lowering of mixing height in autumn, and finally almost no mixing in winter.

**Fig. 6:**
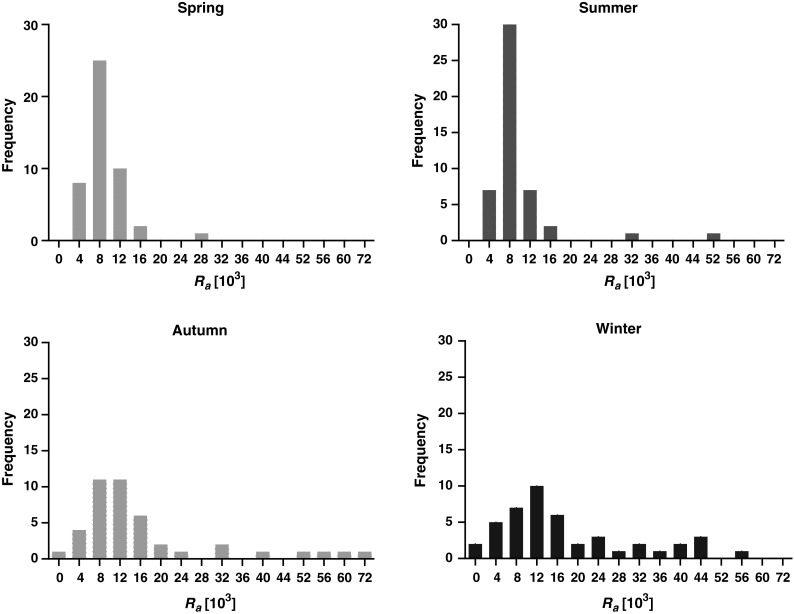
Frequency distribution of ^7^Be/^22^Na activity ratio (*R*_a_) for different seasons over years 2003–2006

**Fig. 7:**
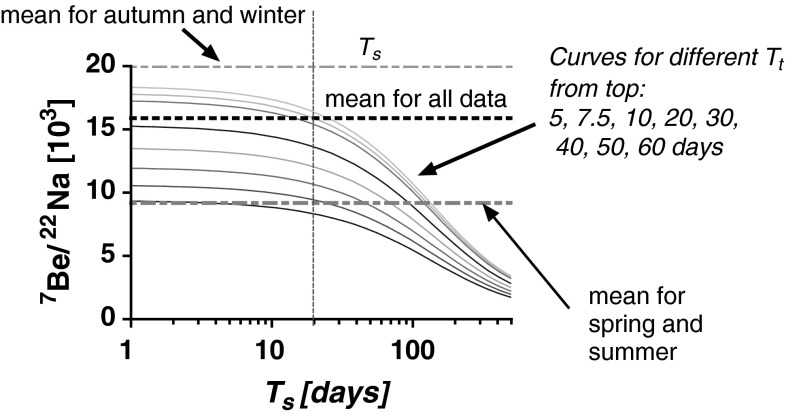
Aactivity ratio ^7^Be/^22^Na (*R*_a_) in ground level air as a function of retention time for aerosols in the stratosphere (*T*_s_). The curves present results of the model given by Eq. 1 [4]. The retention time in the troposphere *T*_t_ can be read out from the picture provided *R*_a_ is known and *T*_s_ assumed

The results presented here allow to adopt a model for aerosol retention developed by Tokuyama and Igarashi [4]. In the original paper the model was applied as follows: the ratio of activity concentration in ground level air ^7^Be/^22^Na (equal to *R*_a_ in Eq. 1) was used to estimate the retention time for aerosols in the troposphere, *T*_t_ (Eq. 1). Since this ratio is season dependable, it should be calculated for each season separately. Frequency distribution for the ^7^Be/^22^Na activity ratio does not take a Gaussian shape, it is rather bimodal or polymodal (see Fig. 6). The data for summer (with high ^22^Na values, so with relatively smaller uncertainties) display the narrowest distribution. Likely, some of the width observed for the frequency distributions comes from the relatively high uncertainties in ^22^Na activity concentration. The mean value for all our results (*n* = 173) is equal to *R*_a_ = (15.9 ± 5.1) × 10^3^, whereas seasonal mean values are shown in Table 3 together with similar results for Japan in 1998. In both countries they were lower for spring and summer (close to 1 × 10^4^) and higher (close to 2 × 10^4)^ for autumn or winter, however the seasonal difference is bigger for Poland which likely results from the lack of autumn maximum of cosmogenic radionuclides reported for Japan (see “Introduction”). Taking into consideration the mean or median value for *R*_a_ and assuming retention time in the stratosphere *T*_s_ equal to 20 days [4] the resulting mean retention time *T*_t_ is equal to about 7.5 days for the entire data set, whereas for warm seasons (when *R*_a_ is close to 1 × 10^4^), with the same *T*_s_ the value of *T*_t_ assumed, it grows to about 50 days (Fig. 7). The cold season with *R*_a_ close to 2 × 10^4^ seems to be well described with this model. *T*_s_ massively longer then 20 days assumed in the original model [4], namely ranging from 102 to 205 days, was obtained recently within the same model in another study [24]. In the presented paper, the *T*_t_ is assumed as known, based on ^210^Po/^210^Pb system, and the model was used to determine *T*_s_. With longer *T*_s_ applied, much shorter *T*_t_ is obtained, far below 10 days as it can be seen from Fig. 7. It does not seem to be very realistic, revealing rather weakness of the applied model.

**Table 3:** Mean values for activity ratio *R*_a_ in different seasons of 2003–2006 obtained for Poland (present work) compared to the results from Japan [4]

	*R*_a_ (mean)				
	All seasons	Spring	Summer	Autumn	Winter
Poland	(15.9 ± 5.1) × 10^3^	11.2 × 10^3^	11.1 × 10^3^	21.3 × 10^3^	18.9 × 10^3^
Japan [4]		(10.0 ± 1.2) × 10^3^	(8.4 ± 1.5) × 10^3^	(8.0 ± 1.2) × 10^3^	(16 ± 4.2) × 10^3^

## Conclusions

Studies on air activity of cosmogenic ^7^Be measured together with ^22^Na and other radionuclides seem to provide a solidly reliable system for studying the atmospheric processes. The applied detection system and sample collection method allowed to measure in weekly collected samples not only ^7^Be, ^137^Cs and ^40^K but also much less active, hence much more difficult to measure, ^22^Na. The determined activities are comparable with those reported by other investigators and show seasonal variation. The increase of cosmogenic radionuclides concentration in air in spring and summer seems to be caused by an injection of stratospheric air followed by the intense mixing of the troposphere. The activity ratio for ^7^Be and ^22^Na allowed us to estimate the retention time for tropospheric aerosols, which appeared to be season dependent: it is longer for warmer seasons (spring, summer), and shorter for colder (autumn, winter) ones. During warmer seasons the temperature gradient between the tropopause and ground level air is much higher, which results in much more intense—in comparison to colder seasons—convectional vertical movements of air masses. This apparently enhances time of aerosol retention in air during warm seasons by nearly an order of magnitude, from less than 10 days as average for all seasons to about 50 days in warm seasons.
